# Special Issue “Volatile Compounds and Smell Chemicals (Odor and Aroma) of Food”

**DOI:** 10.3390/molecules25173811

**Published:** 2020-08-21

**Authors:** Eugenio Aprea

**Affiliations:** 1 Center Agriculture Food Environment, University of Trento/Fondazione Edmund Mach, via E. Mach 1, 38010 San Michele all’Adige (TN), Italy; eugenio.aprea@unitn.it; 2 Department of Food Quality and Nutrition, Research and Innovation Centre, Fondazione Edmund Mach (FEM), via E. Mach 1, 38010 San Michele all’Adige (TN), Italy

**Keywords:** odour, aroma profiling, flavour, food processing, Maillard reaction, oxidation, fermentation, food decay, solid-phase micro-extraction, gas chromatography, spectrometry, gas sensors, fingerprinting, volatilome

Among the constituents of food, volatile compounds are a particularly intriguing group of molecules, because they give rise to odour and aroma. Indeed, olfaction is one of the main aspects influencing the appreciation or dislike of particular food items. Volatile compounds are perceived through the smelling sensory organs of the nasal cavity, and evoke numerous associations and emotions, even before the food is tasted. Such a reaction occurs because the information from these receptors is directed to the hippocampus and amygdala, the key regions of the brain involved in learning and memory.

In addition to identifying the odour-active compounds, the analysis of the volatile compounds in food is also applied for detecting the ripening, senescence, and decay in fruit and vegetables, as well as monitoring and controlling the changes during food processing and storage (i.e., preservation, fermentation, cooking, and packaging). Nineteen research papers and two review papers cover the topics of this Special Issue. The majority of the papers investigated volatile compounds from vegetable origin products (16) and four papers reported volatile compounds from animal origin products (dairy products).

Six of the research papers were mainly focused on the sensory aspects related to volatile compounds [1,2,3,4,5,6], and another six papers investigated aspects related to processing [7,8,9], five papers were more oriented to the quality of the products [10,11,12,13,14], cultivation practice [15] and storage [16,17], two further papers were more focused on measurement techniques and procedures [18,19]. Lastly, the two review papers reviewed the sensory characteristics (flavour and texture) linked to a physiological disorder of apple, namely watercore [20] and the influence of ruminant diet on the volatile flavour compounds in cheese [21].

Muñoz-González et al. [1] studied the effects of ethanol concentration on the dynamics of oral aroma release after wine consumption. They found that a subject’s ethanol concentration and nature of the aroma compound immediately or after 4 min from wine intake influenced oral aroma release. Just after wine intake, a higher release was observed for the more polar compounds and a lower release for more apolar compounds, while after 4 min, all the six esters investigated increased in the oral cavity, increasing ethanol content in wine. The latter effect could be related to an increase in the fruity aroma persistence in the wines.

Endrizzi et al. [2] studied the effect of artificial flavourings on consumer liking of apples. By means of a conjoint experiment, external information (use of claims) was investigated as well. Depending on their personal liking, an apple consumers’ preference is affected by different flavouring treatments. The external information (“traditional” or “selected flavour”) influenced apple acceptability for some groups of consumers depending on their food approach (attitude toward natural food interest and food neophobia).

Deuscher et al. [3] used gas chromatography–olfactometry to classify dark chocolates differing in organoleptic properties by the key aroma compounds. Thirty-eight discriminant key odorants were identified, 13 of which were described for the first time in a cocoa product.

Liberto et al. [4] explored the possibility to use HS-SPME-MS-Enose coupled with chemometrics in order to predict in-cup coffee sensory quality in routine controls. This preliminary study has demonstrated that the proposed approach requires a number of compromises in terms of model robustness and acceptance of the errors in prediction.

In the work of Genovese et al., [5] it was shown that high levels of extravirgin olive oil (EVOO) phenolic compounds influence the in-mouth perceived intensity of both flavours and off-flavours. While the high levels of EVOO phenolic compounds did not produce any effect on the headspace concentration of volatile compounds, they reduced the perceptions of the positive fruity score and of the score of the “fusty–muddy” defect while increased the score of “winey–vinegary” defect.

Yan et al. [6] used fast volatile fingerprinting to differentiate the grades of olive oils (extra virgin, refined and pomace) and indicating the mass peaks more intense for the different olive oils grades.

Fatty acids and their derived volatile compounds were studied in “Thompson Seedless” Raisins during air-drying and sun-drying processes by Wang et al. [7]. The study shows that air-drying is more favourable for the production of fatty acids, whereas sun-drying is more advantageous in terms of fatty acid-derived volatiles.

The work of Ma et al. [8] evaluated the influence of clarification treatments on the volatile composition and aromatic attributes of “Italian Riesling” Icewine. The study shows that while all treatments improved the limpidity of wines, a decreased concentration of aroma compounds was observed as well as a decrease of colour intensity. The aroma and taste properties of the wine samples were influenced more by bentonite fining, while membrane filtration mainly influenced colour and aroma. On the other hand, soybean protein and centrifugation treatments achieved better sensory quality.

Vidal et al. [9] in their study, applied the response surface methodology approach in order to determine the optimum extraction conditions for maximizing the content of volatile compounds and pigments in extra virgin olive oil obtained from Arbequina, Koroneiki, and Arbosana Cultivars. The authors showed that extra-virgin olive oil from irrigated crops and from the Arbequina cultivar had the highest content of volatile compounds.

Chitarrini et al. [10] combined gas chromatography–mass spectrometry and sensory analysis to investigate the aroma of new and standard apple varieties grown at two altitudes. The study indicates twelve volatile organic compounds changing in relationship with pedoclimatic locations, independent of the variety, and significant interactions between variety and altitude on sensory parameters were reported as well.

In a further work, Chitarrini and co-workers [11] used the same approach (gas chromatography–mass spectrometry and sensory analysis) to describe four different honey wines: multi-floral blossom honey and a forest honeydew honey with and without the addition of black currant during fermentation.

Smith and Peterson [12] combined gas chromatography/mass spectrometry/olfactometry with sensory recombination and descriptive analysis to identify aroma differences in refined and whole-grain extruded maize puffs. Maillard reaction products were reported as the main responsible for perceived differences.

Ianni et al. [13] studied the proteolytic process in Caciocavallo cheese obtained from Friesian cows fed zinc, selenium, and iodine supplementation and the possible effect on volatile compounds formation. In particular, it was observed that selenium negatively influenced the biochemical processes leading to the formation of 3-methyl butanol.

A further study on the effect of animal feeding on volatile compounds in dairy products was undertaken by Bennato and co-workers [14]. These authors showed that milk from goats fed a dietary supplementation with olive leaves produced a yogurt, with characteristics potentially indicative of an improvement in nutritional properties and flavour.

Slaghenaufi et al. [15] studied how different modalities of grape withering may influence volatile compounds in young and aged Corvina wines. The fermentative volatile metabolites responsible for wine aroma modulation were affected by the withering process, and in particular, the on-vine withering with blocked xylem is an interesting alternative to conventional fruit withering for the production of wines where a mild withering is requested.

Yang et al. [16] studied the effect of cold storage on the volatile component in jujube fruits by using headspace-gas chromatography–ion mobility spectrometry showing the possibility to determine the different storage periods.

Lobo-Prieto et al. [17] tracked the sensory characteristics of virgin olive oils during storage, showing some disagreements between the sensory assessment and the oxidative stability index analysed by Rancimat.

In the paper of Coelho and co-workers [18], a liquid–liquid microextraction method combined with GC-MS was proposed for the analysis of compounds in fermented beverages and spirits. The method was validated for a set of compounds typically found in fermented beverages.

In the work of Capozzi et al. [19] two complementary analytical techniques, Headspace-Solid Phase Microextraction-Gas Chromatography-Mass Spectrometry and Proton-Transfer Reaction–Mass Spectrometry coupled to a Time of Flight mass analyser were coupled to study the volatile organic profile of Mascarpone cheese. Mascarpone is a base ingredient in industrial, culinary, and homemade preparations (e.g., it is a key constituent of a widely appreciated Italian dessert “Tiramisù”) which has been scarcely investigated for its aroma profile.

Tanaka and co-workers [20], in their review paper, summarized the recent studies related to the physiology of watercore, a physiological disorder of the apple, with special focus on “Fuji” and related cultivars.

Ianni et al., [21] in their review paper, summarized the major families of volatile compounds most commonly found in cheese obtained from lactating dairy ruminants fed experimental diets at various ripening stages, describing, in greater detail, the role of the animal diet in influencing the cheese flavour.

In conclusion, volatile compounds are responsible for the aroma of food and may be influenced by processing, storage, harvesting or animal feeding, and they require a dedicated analytical approach for the identification and the monitoring All these aspects were covered by the manuscripts submitted and published in the Special Issue “Volatile Compounds and Smell Chemicals (Odor and Aroma) of Food”. These manuscripts contributed—with their topics and their quality—to the success of this Special Issue.
